# Human dendritic cell subsets: an update

**DOI:** 10.1111/imm.12888

**Published:** 2018-02-27

**Authors:** Matthew Collin, Venetia Bigley

**Affiliations:** ^1^ Human Dendritic Cell Lab Institute of Cellular Medicine and NIHR Newcastle Biomedical Research Centre Newcastle upon Tyne Hospitals NHS Foundation Trust and Newcastle University Newcastle upon Tyne UK

**Keywords:** antigen presentation/processing, dendritic cell, transcriptomics

## Abstract

Dendritic cells (DC) are a class of bone‐marrow‐derived cells arising from lympho‐myeloid haematopoiesis that form an essential interface between the innate sensing of pathogens and the activation of adaptive immunity. This task requires a wide range of mechanisms and responses, which are divided between three major DC subsets: plasmacytoid DC (pDC), myeloid/conventional DC1 (cDC1) and myeloid/conventional DC2 (cDC2). Each DC subset develops under the control of a specific repertoire of transcription factors involving differential levels of IRF8 and IRF4 in collaboration with PU.1, ID2, E2‐2, ZEB2, KLF4, IKZF1 and BATF3. DC haematopoiesis is conserved between mammalian species and is distinct from monocyte development. Although monocytes can differentiate into DC, especially during inflammation, most quiescent tissues contain significant resident populations of DC lineage cells. An extended range of surface markers facilitates the identification of specific DC subsets although it remains difficult to dissociate cDC2 from monocyte‐derived DC in some settings. Recent studies based on an increasing level of resolution of phenotype and gene expression have identified pre‐DC in human blood and heterogeneity among cDC2. These advances facilitate the integration of mouse and human immunology, support efforts to unravel human DC function *in vivo* and continue to present new translational opportunities to medicine.

## Introduction

**Figure 1:**
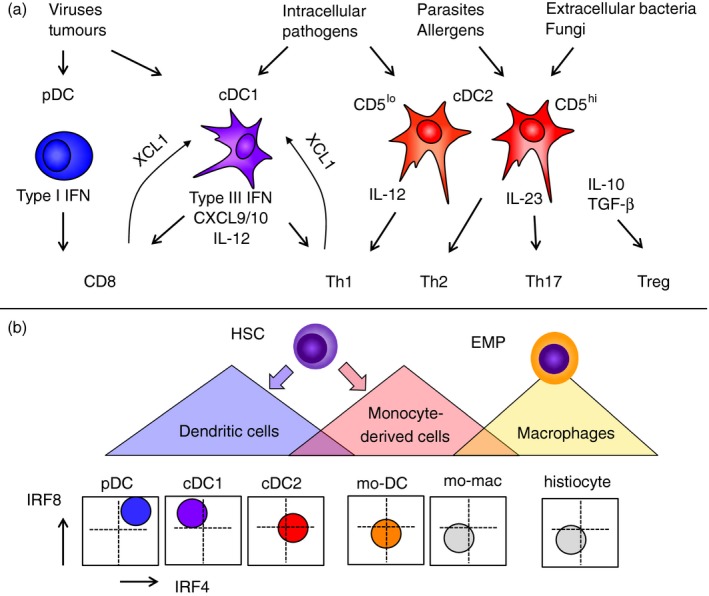
Ontological overview and functional specialization of human dendritic cells (DC). (a) DC are often depicted as a single ‘all purpose’ cell in diagrams of T‐cell differentiation but each subset is specialized to make specific responses to pathogen or danger signals. Depending on the context, many different responses may be observed and selected principal functions of human plasmacytoid DC (pDC), conventional DC1 (cDC1) and cDC2 are depicted. (b) Ontological basis of DC, monocyte and macrophage classification. Haematopoietic stem cells (HSC) give rise to DC and monocyte‐derived cells by distinct routes marked by differences in the relative expression of interferon regulatory factor 8 (IRF8) and IRF4 as shown in schematic bivariate plots beneath. Monocytes are IRF4/8 low but can be induced to differentiate into monocyte‐derived DC (mo‐DC). Monocyte‐derived macrophages are also ontologically distinct from many populations of long‐lived resident macrophages derived from early myeloid progenitors (EMP).

The initiation and control of immune responses depends upon dendritic cells (DC), a class of bone‐marrow‐derived cells found in blood, tissues and lymphoid organs. In a broad sense, the function of DC is to bridge the innate and adaptive immune systems. DC are innate immune cells because they recognize and respond to pathogen‐associated and danger‐associated signals, shaping the acute inflammatory response. Their defining role in adaptive immunity is to process extracellular and intracellular proteins and to present antigens in the context of MHC molecules to prime naive T cells. Previously, DC have been characterized as universal ‘all purpose’ antigen‐presenting cells, but an important aspect of the control of immune responses, is the existence of several different types of DC, each specialized to respond to particular pathogens and to interact with specific subsets of T cells. This expands the flexibility of the immune system to react appropriately to a wide range of different pathogens and danger signals (Fig. 1a).

## The unified classification of mammalian DC

The functional and anatomical classification of DC as ‘migratory tissue DC’ or ‘lymph node resident DC’ remains useful to define context. However, comparative gene expression studies have driven a robust classification of DC based primarily on lineage, that correlates with the differential expression of key transcription factors such as interferon regulatory factors 8 and 4 (IRF8 and IRF4) and performs well across all mammalian species.^1^, ^2^, ^3^, ^4^ This recognizes plasmacytoid DC (pDC) and two types of ‘conventional’ or ‘classical’ DC (cDC) corresponding to the two subsets of myeloid DC previously defined by CD141 and CD1c expression in humans.^5^, ^6^, ^7^ These DC lineage cells are treated as distinct entities to monocyte‐derived cells and monocyte‐derived DC (mo‐DC) and macrophage populations derived from primitive myeloid progenitors arising in the yolk sac (Fig. 1b).

The term ‘myeloid’, introduced into human DC biology around two decades ago,^5^, ^6^, ^7^ remains valid in defining a common set of antigens found on cDC and for clarity, ‘myeloid’ and ‘conventional’ will be used together to describe the human DC of this class. The original markers, CD141 and CD1c have limitations as both are induced on cDC and monocyte‐derived cells in tissues and in culture. Expression profiling has now provided a suite of more consistent markers that perform well across species, such as CLEC9A, CADM1, BTLA and CD26 for CD141^+^ myeloid cDC1, and CD2, Fc*ε*R1 and SIRPA for CD1c^+^ myeloid cDC2^2^, ^3^, ^4^ (Table 1). Additional complexity discussed below is the emergence of subsets of cDC2 and the realization that conventional pDC gates include myeloid cDC precursors that also express CD123 and CD303.^8^, ^9^, ^10^

**Table 1:** Human dendritic cell subset characterization

Unified classification	Differential TFs	Conventional markers	Extended markers	Notes
Plasmacytoid DC	E2‐2	CD123,	FCER1	DC6 9
	ZEB2	CD303/CLEC4C/BDCA‐2	ILT3, ILT7	
	**IRF8**	CD304/NRP1/BDCA‐4	DR6	
	**IRF4**			
Myeloid cDC1	ID2	CD141/BDCA‐1	CLEC9A	DC1 9
	**IRF8**		CADM1	No antibody for XCR1 in human
	BATF3		XCR1	
			BTLA	
			CD26	
			DNAM‐1/CD226	
Myeloid cDC2	ID2	CD1c/BDCA‐1	CD2	DC2/DC3 9
	ZEB2	CD11c	FCER1	DCIR clone specific [26]
	**IRF4**	CD11b	SIRPA	
	Notch2/KLF4		ILT1	
			DCIR/CLEC4A	
			CLEC10A	
Langerhans cell	ID2	CD207	EpCAM	
	RUNX3	CD1a	TROP2	
		E‐Cadherin		
Pre‐DC	ZEB2	CD123, CD303	AXL	DC5 ‘AS’ DC 9
	**IRF4**		SIGLEC 6	
	KLF4		CX3CR1	
			CD169 (SIGLEC 1)	
			CD22 (SIGLEC 2)	
			CD33 (SIGLEC 3)	
Mo‐DC	MAFB	CD11c	SIRPA	
	KLF4	CD1c/BDCA‐1	S100A8/A9	
		CD1a	CD206	
			DC‐SIGN/CD209	
Non‐classical monocyte		CD16		DC4 9
		CX3CR1		SLAN DC?
		+/‐SLAN		

cDC, conventional DC; DC, dendritic cell; Mo‐DC, monocyte‐derived DC; pDC, plasmacytoid DC; TF, transcription factor. IRF4 and IRF8 are highlighted in bold.

## Analysis of human dendritic cells, monocytes and macrophages

Fluorescence flow cytometry is the most commonly used platform for identifying human DC.^2^, ^3^, ^4^ This has been expanded from the analysis of blood, to single‐cell suspensions of tissues including skin,^11^, ^12^, ^13^ lung^14^, ^15^, ^16^ intestine^17^ and liver of humans^2^, ^18^, ^19^ and to lymphoid tissue^3^, ^4^, ^20^, ^21^, ^22^ fetal tissues^23^ and other body fluids.^24^

The Tenth Human Leucocyte Differentiation Antigen workshop has recently reported a range of new antibodies characterized on human DC populations (Table 1).^25^, ^26^ Advances through cytometry by time‐of‐flight now enable 30–40 antigens to be analysed simultaneously, facilitating the dissection of complex populations of leucocytes or ‘deep phenotyping’ of selected lineages. A number of computational flow cytometry tools have been developed to scale and represent high dimensional data such as FlowSOM, tSNE, oneSENSE^2^, ^23^ and ISOMAP.^10^ This approach, rather than sequential manual gating, aids the unbiased mapping and discovery of new cell phenotypes from multiparameter data, including that generated by conventional fluorescence flow cytometry (reviewed in ref. 27).

In recent years, transcriptomics has moved rapidly from expression arrays of bulk populations to single cell RNAseq.^9^, ^10^ These studies provide novel surface markers, reveal heterogeneity within subsets and identify small but critical DC precursor populations.^9^, ^10^ It is noteworthy that such unbiased approaches support the major classification of known populations of pDC, cDC1 and cDC2 and confirm that empirically defined markers such as CLEC9A and CD1c are the most highly discriminatory according to formal computation.^9^, ^10^ Using oligonucleotide tags, it is also possible to combine antibody‐based phenotyping with transcriptomics.^28^

## New models of haematopoiesis and the origin of human DC

Dendritic cells have a finite lifespan of days to weeks after entering the periphery and must be continually replenished by haematopoiesis. The use of media containing Flt3 ligand, stem cell factor and granulocyte–macrophage colony‐stimulating factor (GM‐CSF; ‘FSG’) with murine stromal cells such as MS5 permits the generation of pDC and cDC1 that are easy to identify as genuine DC with no relationship to mo‐DC.^29^, ^30^, ^31^, ^32^ It is more challenging to distinguish between myeloid cDC2 and DC derived from monocytes, that form naturally in these cultures, but retention of CD14 has been used as a *de facto* marker of likely monocyte origin.^9^, ^10^, ^32^

**Figure 2:**
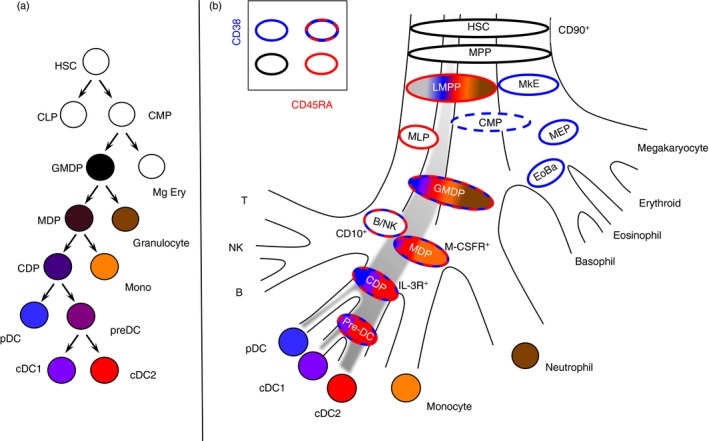
Classical and revised models of human haematopoiesis. (a) In classical models of haematopoiesis, cell potential partitions by successive bifurcations descending from the apex where common lymphoid and common myeloid progenitors (CLP; CMP) arise from the haematopietic stem cell (HSC). Each progenitor population has homogeneous differentiation potential such that every cell has an equal probability of two mutually exclusive fates. Hence, dendritic cells (DC) were proposed to arise in the sequence: CMPs, granulocyte–macrophage DC progenitor (GMDP), macrophage DC progenitor (MDP), common DC progenitor (CDP) with a final pre‐DC stage leading to conventional DC1 (cDC1) and cDC2. Each population is given a uniform colour to indicate homogeneous potential. (b) Experimental data support several revisions to the classical model. First lineage is primed in early progenitors so that most populations contain only cells with a single potential. Second, lymphoid and myeloid potential run together originating as the lymphoid primed multi‐potent progenitor (LMPP) that separates from megakaryocyte and erythroid potential (MkE) at the apex. Hence the gates defined by CD38 (blue borders) and CD45RA (red borders) contain phenotypically related cells but with restricted potentials, indicated by bands of colour each corresponding to a discrete lineage.

Recent conceptual revolutions in haematopoiesis have had a profound impact upon models of DC ontogeny. First, the existence of a hierarchy of multipotent progenitors that make a series of dichotomous fate decisions (Fig. 2a), has been replaced by the notion that each progenitor follows a predestined pathway according to lineage priming that occurs at early stages in development (Fig. 2b). In experimental terms, this means that a phenotypically defined population does not contain a homogeneous population of multi‐potent cells, but rather, a cross‐section of cells primed by related but distinct developmental pathways that share a common, transient phenotype.^33^, ^34^, ^35^, ^36^ Entities such as the macrophage–dendritic cell progenitor (MDP) and common dendritic cell progenitor (CDP) are evanescent. Although bi‐potential and tri‐potential cells exist, profiling of > 2000 clonal outputs from the entire range of human progenitors does not find any significant populations corresponding to human MDP or CDP.^32^ Regions thought to contain such multi‐potent cells mostly comprise phenotypically related cells with a single potential.

Second, the classical dichotomy between lymphoid and myeloid lineages, placed at the apex of haematopoiesis, has been thoroughly revised. Common myeloid progenitors are mixtures of mega‐erythroid and myeloid precursors and the most significant early partitioning of cell fate occurs when megakaryocyte and erythroid potentials separate from lympho‐myeloid potential.^33^, ^34^, ^37^ In contemporary models, lymphoid‐primed multipotent progenitors are at the apex of all myeloid and lymphoid lineages.^34^, ^36^ The important consequence of this is that it is no longer necessary to puzzle over the apparent ‘dual’ lymphoid and myeloid origin of DC, because DC are a product of the core lympho‐myeloid pathway in which both traits may be expressed by emerging progeny.

**Figure 3:**
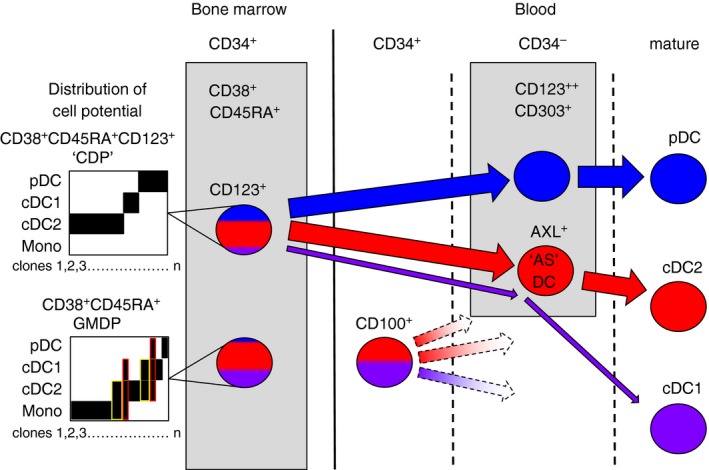
Segregation of human dendritic cell (DC) potential in late precursor compartments. The CD34^+^ CD38^+^ CD45RA^+^ human granulocyte–macrophage DC progenitor (GMDP) contains only a minority of progenitor cells with bi‐ or tri‐potential indicated in yellow and red, respectively in the diagrams of cell potential of several hundred individual progenitors differentiated *in vitro* (schematic redrawn from data of Lee *et al*.^32^). A small minority of progenitors in the GMDP qualify as MDP [except without plasmacytoid DC (pDC) potential] or common DC progenitor (CDP; all three DC potentials). The CD123^+^ fraction of GMDP has been described as a CDP; although it does not contain monocyte potential, trilineage common DC progenitors are not found in this gate. In the blood, a CD34^+^ CD123^−^ precursor fraction is found to contain conventional DC1 (cDC1) and cDC2 potential^9^ together with a CD34^–^ CD123^+^ AXL^+^ SIGLEC6^+^ pre‐DC with mainly cDC2 potential.^9^, ^10^ Traces of cDC1 potential are also found in CD123^+^ cells. It is not known how the CD34^+^ CD123^−^ blood precursor relates to GMDP, if it is a physiological route of cDC differentiation, or whether this occurs via AXL^+^ precursors. Broken lines indicate unconfirmed relationships.

Hence pDC, cDC1 and cDC2 potential can be traced through all the previously defined human progenitor compartments from haematopoietic stem cells, through lymphoid‐primed multipotent progenitors to portions of the granulocyte macrophage DC progenitor (GMDP) with either high CD115 expression (MDP‐like) or high CD123 expression (CDP‐like) that contain mainly uni‐potent progenitors for each DC lineage^32^ (Fig. 3). Where DC are derived from two different regions of the CD34^+^ compartment, they emerge transcriptionally homogeneous, illustrating the importance of intrinsic regulatory circuits in defining lineage and the limitations of phenotyping in identifying discrete potentials.^31^

Another aspect of the lympho‐myeloid model of haematopoiesis is that DC can be ordered in a spectrum of phenotypes from lymphoid to myelo‐monocytic that mirrors their dominant developmental pathway.^32^ It has been known for many years that pDC are most ‘lymphoid’ harbouring RAG and rearranged IgH loci^38^, ^39^ and expressing many markers that are distinct from monocytes. Myeloid cDC1 still express a mixture of unique markers that separate them from monocytes in addition to myeloid antigens that connect them with myeloid lineages. At the other end of the spectrum, myeloid cDC2 share the most markers with monocytes and can be difficult to dissociate entirely from mo‐DC.

## Human pre‐dendritic cells and AXL^+^SIGLEC6^+^ DC

Between CD34^+^ progenitors and mature DC, there exist potential pre‐DC defined as DC‐restricted precursors that do not yet express the full phenotype of mature DC. Several groups have reported human pre‐DC that fulfil these criteria although it is not yet completely clear how they relate to one another. Breton and colleagues focused on CD34‐negative myeloid cDC precursors, excluding pDC with CD123 and CD303, cDC1 with CD141 and cDC2 with CD1c^40^ before selecting cells with expression of the growth factor receptors CD117 (c‐kit), CD116 (GM‐CSF receptor) and CD135 (Flt‐3). Other investigators have found more numerous populations that fit the definition of pre‐DC by examining every lineage‐negative HLA‐DR‐positive cell using single‐cell transcriptomics^9^ or a combination of deep phenotyping and single‐cell trancriptomics.^10^ The critical advantage of these recent studies is that they did not begin by excluding mature DC and so found many pre‐DC among the CD123^+^ populations that had been excluded as mature pDC by Breton and colleagues. CD123 has long been used as a marker to identify pDC but both new studies emphasize the critical point that myeloid cDC‐like cells are also captured in CD123^+^ populations (Fig. 3). Moreover, the inadvertent inclusion of these cells has confounded many previous studies of pDC and explains the observations that pDC can apparently differentiate into myeloid cDC *in vitro*.^41^ A re‐interpretation of these results would suggest that *in vitro* culture causes short‐lived mature pDC to decline, while differentiating myeloid cDC come to dominate the preparation. This conclusion had been anticipated to a degree by a number of reports describing subsets of CD123^+^ pDC marked by CD2 or CD56 that show myeloid DC characteristics of inferior type I interferon production, higher interleukin‐12 (IL‐12) production and greater allo‐stimulatory capacity.^42^, ^43^, ^44^, ^45^, ^46^

CD303/CLEC4C and CD304/NRP1 overlap in expression between the myeloid cDC precursor component and pDC and cannot be used to separate the two populations completely, although the highest expressing cells will include only pDC.^9^ The non‐pDC cells begin to express myeloid cDC antigens such as CD11c, CD33 (SIGLEC 3) and CX3CR1.^10^ AXL and SIGLEC 6 (CD327) emerge as particularly useful, leading Villani and colleagues to define a new ‘AS’ DC subset (DC5 in their classification) (Table 1). In favour of AS DC being a distinct functional entity they stimulate T cells vigorously and are found in lymphoid tissue. As potential pre‐DC, the authors note that they ‘exist in a continuum of states *in vivo* with the potential to transition towards cDC2’.^9^ Intrinsic DC function or precursor status are not mutually exclusive roles and AS DC may be immediately recruited for function by some stimuli or undergo maturation to cDC2 in response to others (as yet to be defined). See *et al*.^10^ prefer to characterize a population containing AXL^+^ SIGLEC6^+^ cells primarily as precursors, describing them as ‘early pre‐DC’ with the ability to develop into cDC1 and cDC2. In support of this, See *et al*. observe that the ratio of cDC1:cDC2 production by their pre‐DC is in proportion with that of mature cells in the blood. Taking a slightly wider population of CD33^+^ CD45RA^+^ CD123^+^ cells, these authors also describe lineage priming in pre‐DC beginning to express CADM1 (pre‐cDC1) and CD1c (pre‐cDC2).

By further relaxing the gating to include CD34^+^ cells, Villani and colleagues identified another DC‐restricted precursor in human blood described as CD34^+^ CD100^+^ (Fig. 3). Intriguingly, this cell has lower expression of CD123 than AXL^+^ SIGLEC 6^+^ pre‐cDC and appears to be more primitive, owing to its lack of CD116 (GM‐CSF receptor) and higher proliferative capacity.^9^ It has approximately equal cDC1 and cDC2 potential *in vitro* and has the highest enrichment of pre‐cDC1 potential in human blood so far described. CD34^+^ CD100^+^ cells potentially emerge directly from a compartment of the GMDP but further experiments will be required to test this and also to map any relationship with the AXL^+^ pre‐cDC.

## Plasmacytoid dendritic cells

### Phenotype and distribution

**Figure 4:**
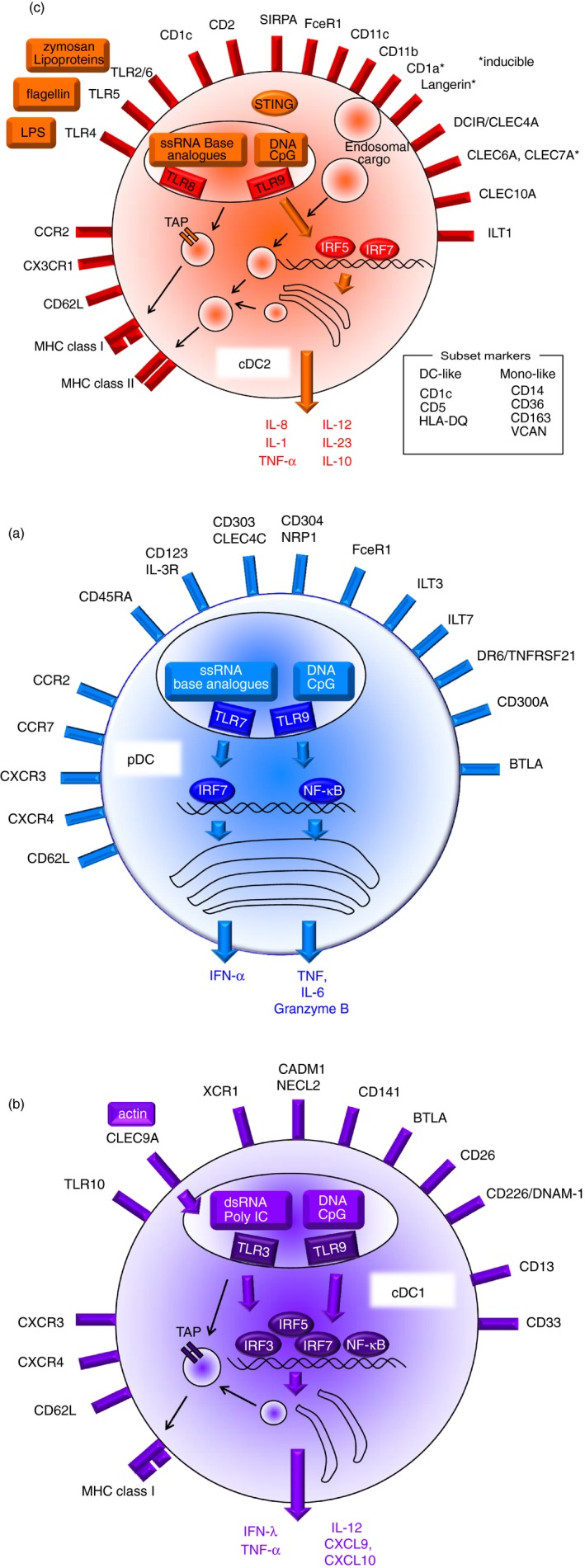
Features of the principal human dendritic cell (DC) subsets. Diagrams of the main surface markers, pathogen sensors and responses of (a) plasmacytoid DC (pDC); (b) conventional DC1 (cDC1) and (c) cDC2. Data are principally drawn from observations on freshly isolated blood DC and do not capture the full range of responses possible following inflammatory activation.

Plasmacytoid dendritic cells have an eccentric nucleus and prominent endoplasmic reticulum and golgi (resembling a plasma cell) for the production of type I interferon (Fig. 4a). They were first identified in human blood and tonsil.^41^, ^47^, ^48^ Unlike myeloid cDC, they do not express the myeloid antigens CD11c, CD33, CD11b or CD13.^5^, ^6^, ^49^ They retain expression of the GMDP markers CD123 (IL‐3R) and CD45RA, which are down‐regulated when DC progenitors differentiate into myeloid cDC. Like all human DC they express CD4, at a higher level than myeloid cDC.^50^ In addition, pDC have an array of surface receptors that are intimately involved in the regulation of their major physiological function, the production of type I interferon. These include the well‐known human pDC markers CD303 (CLEC4C; BDCA‐2), CD304 (neuropilin; BDCA‐4) CD85k (ILT3) and CD85g (ILT7) together with more recently characterized antigens Fc*ε*R1, BTLA, DR6 (TNFRSF21/CD358) and CD300A.^51^, ^52^ Transciptional profiling has also added the markers FAM129C, CUX2 and GZMB.^3^ Several recent papers have described a small subset of CD123^+^ pDC that express CD2^+^,^42^, ^43^ CD56^+^ 44, 45 or CD5.^46^ These cells have a distinct gene expression pattern that overlaps with myeloid cDC and are now known to contain AXL^+^ SIGLEC 6^+^ myeloid pre‐cDC as described above. The two populations do not completely overlap; some AXL‐negative pDC appear to express CD2 or CD5^43^, ^46^ and CD56 cannot be evaluated in the single‐cell studies because it was excluded in lineage. However, studies seeking to define the characteristics of ‘pure’ pDC should exclude contamination with myeloid pre‐DC using AXL and other markers.^9^, ^10^

### Development

Dendritic cell development in mammals is dependent on the coordinated action of many transcription factors that facilitate lympho‐myeloid differentiation including GATA2, PU.1, GFI1, IKZF1 and IRF8^53^, ^54^, ^55^, ^56^, ^57^ (reviewed in refs. 58, 59, 60). Heterozygous GATA2 mutation and bi‐allelic IRF8 mutation abrogate pDC development in humans.^61^, ^62^ Humans with an IKZF1 mutation have a selective pDC deficiency EcDC1 production.^63^

A key axis in regulating the balance of pDC and myeloid cDC development is the antagonism between ID2, an inhibitor of DNA binding, and E2‐2, a basic hemophagocytic lymphohistiocytosis protein.^58^, ^60^ E2‐2 is a lineage‐determining factor for pDC that is negatively regulated by ID2.^64^ Several recent reports describe transcription factors that influence the relative production of pDC and cDC, through interaction with this axis. The ETO family protein MTG16 and the transcription factor ZEB2 are reported to repress ID2, so increasing pDC production,^65^, ^66^, ^67^ while NFIL3 acts to reduce pDC in favour of cDC1 production.^68^ Exogenous growth factors GM‐CSF (acting through STAT5) and Flt3L (acting through STAT3) respectively inhibit or enhance pDC development by modulating the expression of ID2 and E2‐2. SPIB and BCL11A also positively regulate the pDC lineage through early commitment and enhanced survival. Downstream targets of E2‐2 include SPIB and BCL11A, so stabilizing the lineage through positive feedback. E2‐2 also promotes many factors critical to the function of pDC including IRF7 and IRF8, RUNX2 and CIITA (reviewed in refs. 58, 59, 60). In humans, heterozygous mutation or loss of E2‐2 causes Pitt–Hopkins syndrome in which mature interferon‐*α* (IFN‐*α*)‐secreting pDC are reduced in number. Accurate exclusion of pre‐cDC in the analysis of this syndrome reveals a greater pDC deficiency than previously suspected,^10^ although this does not cause overt immunodeficiency.

### Functions and role in immunity

Plasmacytoid DC are specialized to sense and respond to viral infection through several mechanisms by the rapid production of high quantities of type I and type III interferons and secretion of cytokines.^52^, ^59^ Toll‐like receptor 7 (TLR7) and TLR9 are the key endosomal pattern recognition receptors that sense single‐stranded RNA and double‐stranded DNA, respectively.^52^ STING has also been reported as playing a role in DNA sensing.^69^ Depending upon the nature of the nucleic acid cargo and mode of delivery, interferon and cytokine production may be differentially regulated.^70^ IRF7 is the major transducer of type I interferon production in pDC,^71^ whereas production of tumour necrosis factor and IL‐6 is dependent upon the nuclear factor‐κB pathway. Many other signalling molecules participate in and regulate this process including MyD88 and DOCK2 (reviewed in refs. 52, 59).

Ligation of surface receptors modulates activation or tolerance through the regulation of the IRF7 and nuclear factor‐κB pathways. CD300A transmits enhancing signals through an ITIM domain^51^ while ligation of Fc*ε*R1, ILT7 and CD303 (BDCA2) inhibits IFN‐*α* production via ITAM signalling (reviewed in ref. 52). Death Receptor 6 (DR6; TNFRSF21/CD358) is a specific marker of human pDC and knock‐down in pDC cell lines impairs IRF7 translocation to the nucleus.^72^ Sphingosine‐1‐phosphate signalling interacts with ILT7 to limit TLR‐induced interferon production^73^ and with IFNAR to terminate the IFN‐*α* response.^74^

The importance of IRF7 in regulating IFN‐*α* production in humans was underscored by the severe susceptibility to influenza and demonstrable lack of IFN‐*α* production by pDC in a patient with compound heterozygous IRF7 mutation.^75^ Deficiency of MyD88 and IRAK4 are predicted to affect pDC whereas DOCK8 deficiency is known to be associated with decreased pDC number and function.^76^ In addition to acute viral infection, pDC have been studied in chronic infections. Early production of IFN‐*α* by gut pDC appears to be beneficial in HIV elite controllers^77^ but in chronic hepatitis, persistence may be promoted by attenuated pDC responses and pDC‐mediated induction of T‐cell tolerance.^78^, ^79^ There is scope for more detailed analysis of the role of pDC in viral infections and even more so for bacterial and fungal pathogens. The prominent role of type I interferon production and signalling and potential of pDC to sense self‐nucleic acids^80^ have implicated pDC in the pathogenesis of psoriasis,^81^ systemic lupus erythematosus^82^ and other autoimmune diseases. Many potential therapeutic targets are presented by the factors that modulate IFN‐*α* release by pDC.

Direct targeting of antigens to pDC through CD303 (CLEC4C; BDCA‐2)^49^ or CD367 (CLEC4A, DCIR)^83^, ^84^ suggests that pDC are capable of priming CD4 T cells. *In vitro* experiments have also measured cross‐presentation to CD8 T cells.^85^, ^86^ However, some pDC preparations used in functional studies may have been contaminated with myeloid cDC precursors with superior ability to process and present antigens to T cells^9^, ^10^ and further evaluations of the antigen‐presenting capacity of pDC are warranted.

Conflicting roles for pDC have been reported in allergy.^87^, ^88^ Tolerogenic pDC under the influence of GM‐CSF have also been proposed to contribute to tumour progression.^89^

## IRF8/BATF3‐dependent myeloid cDC1

### Phenotype and distribution

Human myeloid cDC1 are present at approximately one‐tenth the frequency of cDC2 in steady‐state blood and tissues.^2^, ^3^, ^4^, ^7^, ^12^ They were originally described as a subset of blood DC with high expression of CD141^+^ (BDCA‐3, thrombomodulin)^5^, ^6^ (Fig. 4b). In common with myeloid cDC2, they express CD13 and CD33, but differ by low CD11c and little CD11b or SIRP*α* (CD172). Care must be taken not to exclude them by selecting myeloid cDC with only high CD11c expression. CD141 alone is not a reliable marker because monocytes and CD1c^+^ cDC2 acquire a moderate level of expression in tissues and *in vitro*.^12^, ^90^ Other markers should be used for confirmation: CLEC9A, the receptor for actin exposed during cell necrosis,^91^, ^92^ the cell adhesion molecule CADM1 (NECL2) and the antigen BTLA considerably increase the accuracy of identification. Indoleamine 2,3‐dioxygenase is also highly expressed.^3^ Lack of expression of monocyte and cDC2 markers such as CD14, CD11b and SIRP*α* is also important. XCR1 is also a conserved marker in many species, as identified by several gene expression studies.^93^ Monoclonal antibodies have been harder to develop because the structure is highly conserved, but hybrid cytokine‐Fc reagents have been successfully used.^2^ Intracellular detection of IRF8 may be considered a reference standard for identifying this lineage because unopposed expression of IRF8 (without IRF4) defines the lineage.^2^ However, intracellular staining prevents many subsequent transcriptomic and functional tests from being performed.

Human cDC1 are found in blood and among resident DC of lymph node, tonsil, spleen and bone marrow^3^, ^4^, ^20^, ^29^, ^94^, ^95^ and non‐lymphoid tissues, skin, lung, intestine and liver.^2^, ^3^, ^4^, ^14^, ^15^, ^16^, ^17^, ^18^, ^19^ There are suggestions that they are more enriched in tissues than in the blood, although this has not always been backed up by sufficiently accurate characterization.

### Development

Myeloid cDC1 development is dependent upon GATA2, PU.1, GFI1, Id2, IRF8 and Basic leucine zipper transcription factor (BATF3)^53^, ^54^, ^55^, ^56^, ^96^, ^97^, ^98^ (reviewed in refs. 58 and 60). IKZF1 deficiency in humans ablates pDC but results in an increase in cDC1.^63^ IRF8 acts to preserve DC potential at several points in haematopoiesis by direct or indirect competition with a series of transcription factors that promote other lineages: (i) IRF8 limits CEBPA‐mediated granulocytic differentiation; (ii) with PU.1 it interacts with KLF4 to modulate the balance of DC to monocyte differentiation; (iii) it competes with IRF4 to control cDC1:cDC2 output and; (iv) a BATF3 ‘switch’ ensures that unopposed IRF8 maintains cDC1 maturation.^99^ Gene dosage is a critical determinant in understanding the effect of IRF8 variants upon DC development. This has been thoroughly explored in the mouse through targeted hemizygous and homozygous deletion^100^ but becomes even more complex when amino acid substitutions are considered, as in human examples of IRF8 variation.^61^, ^62^ Generally speaking, more severe losses of IRF8 activity incur earlier defects in haematopoiesis. Hence homozygous deletion causes excess production of neutrophils and loss of monocytes and all DC.^61^, ^62^ At the other extreme, subtle losses of IRF8 activity may only affect the production of cDC1, as documented in BXH2 mice carrying the IRF8 hypomorphic allele R294C and IRF8 hemizygous mice.^100^, ^101^ Other lineages are also affected by IRF8 mutation including B cells and natural killer cells, and human phenotypes show variant‐specific idiosyncracies.^61^, ^62^, ^102^ IRF8 is expressed at a low level in haematopoietic stem cells and early progenitors. It has been suggested that the ratio of IRF8 to PU.1 determines DC lineage potential from primitive stages of haematopoiesis.^32^ The multi‐level regulation of DC‐poiesis by IRF8, in concert with interferon‐mediated signalling and other pathways such as wnt/*β*‐catenin and notch, is likely to modulate cellular output during inflammatory stress.^103^ In humans, short hairpin RNA knock‐down of BATF3 prevents cDC1 maturation *in vitro*.^104^ Immune activation of other BATFs is able to bypass BATF3 deficiency in mice, suggesting another pathway in which inflammation may increase the flux of cDC1 development.^105^

### Functions and role in immunity

Myeloid cDC1 have been characterized as a subset of DC that have a high intrinsic capacity to cross‐present antigens via MHC class I to activate CD8^+^ T cells and to promote T helper type 1 (Th1) and natural killer responses through IL‐12.^12^, ^29^, ^94^, ^106^ Cross‐presenting capacity per se is less restricted to the cDC1 subset in humans than in mouse,^20^, ^21^, ^107^, ^108^, ^109^ as reflected by lower enrichment for MHC class I presentation pathway genes than in murine cDC1.^110^, ^111^

Human cDC1 secrete surprisingly low IL‐12 compared with appropriately activated cDC2 or mo‐DC.^12^, ^20^, ^107^ This has been a subject of some controversy but is in line with the observation that human cDC2 and mo‐DC also have significant ability to interact and present antigens to Th1 cells.^107^, ^109^, ^112^ It appears that both cross‐presentation to CD8^+^ T cells and Th1 activation are less restricted to the cDC1 lineage in humans than in mice.

These differences aside, human cDC1 possess several conserved mechanisms that mediate efficient recognition of viral and intracellular antigens, transport of antigen to the appropriate endosomal compartments^108^ and production of type III interferon. They are also intrinsically resistant to productive viral infection.^113^ CLEC9A, a key marker of cDC1, is a unique receptor that recognizes bare actin filaments exposed upon necrotic cell death^91^, ^92^ and directs cell‐associated antigens into the cross‐presentation pathway.^114^, ^115^ Myeloid cDC1 express TLR3, TLR9 and TLR10 and among DC, TRL3 and TLR10 are selectively expressed.^9^, ^116^, ^117^ TLR3 plays an important role in the recognition of dsRNA and production of type I interferons via IRF3.^118^ Defects in the TLR3/IRF3 axis in humans are associated with specific susceptibility to HSV1 encephalitis through attenuated responses to viral RNA, although not necessarily uniquely mediated by cDC1.^119^ TLR9 is less well studied in cDC1 but is potentially also important in interferon responses to DNA, as in pDC. Myeloid cDC1 are also major producers of type III interferons IFN*λ*1–3^120^ and their accumulation in hepatitis C virus infection has been linked to the beneficial role of type III interferon in viral clearance.^18^

Expression of the XCR1 chemokine receptor is another conserved feature of cDC1 that enables close interaction with XCL‐producing activated T cells and natural killer cells. Several recent studies indicate the importance of this axis in coordinating peripheral Th1 and cytotoxic responses^121^, ^122^ in reciprocity with the action of DC‐derived CXCL9/10 upon activated T cells.^123^

In mice, cDC1 have also been characterized as cross‐priming tolerogenic cells but this potential is not well documented in humans.^124^, ^125^ In this respect, the restricted expression of TLR10 by cDC1 is intriguing. It has no known ligand and no mouse homologue but has recently been shown to be a negative regulator of TLR signalling.^126^ CD141 is also thought to transmit negative regulation.^127^

## IRF4/KLF4/NOTCH2‐dependent myeloid cDC2

### Phenotype and distribution

The major population of myeloid cDC in human blood, tissues and lymphoid organs are characterized as myeloid cDC2 expressing CD1c, CD2, FcεR1, SIRPA and the myeloid antigens CD11b, CD11c, CD13 and CD33 (Fig. 4c). Recent transcriptional profiling has identified CLEC10A (CD301a), VEGFA and FCGR2A (CD32A) as consistent cDC2 markers, together with the lack of cDC1 markers.^3^ In tissues, dermal cDC2 were first characterized as migratory CD1a^+^ CD1c^+^ DC.^128^, ^129^ CD1a is easily acquired by cDC2 but neither CD1a nor CD1c are completely restricted to cDC2 and may be expressed by cDC1 and mo‐DC isolated from tissues or in culture.^12^, ^130^

In the skin, cDC2 may be distinguished from Langerhans cells (LC) by higher CD11c and CD11b but lower CD1a, Langerin and EpCAM.^22^, ^131^ Notably, tissue cDC2 spontaneously express low langerin,^22^, ^131^, ^132^ in contrast to mouse in which cDC1 express langerin. Furthermore, blood cDC2 can be induced to express high langerin and Birbeck granules in response to transforming growth factor‐*β* (TGF‐*β*) *in vitro* 133, 134 although it remains unknown whether cDC2 have any potential to form LC *in vivo*. Myeloid cDC2 have also been described in the lung,^14^, ^15^, ^16^ intestine^17^ and liver of humans.^2^, ^4^, ^19^ In the lymph node, most interdigitating cells of the T‐cell areas have a phenotype compatible with cDC2 lineage.^3^, ^4^, ^21^, ^135^, ^136^, ^137^ Tonsil and spleen also contain CD1c^+^ DC.^20^, ^29^, ^138^ As these tissues do not receive afferent lymph, it is concluded that some CD1c^+^ DC are ‘resident DC’ originating directly from the blood.

Detailed phenotyping and single‐cell gene expression studies have recently characterized two subsets of cDC2 in human blood, one ‘DC‐like’ with higher expression of CD5, CD1c, HLA‐DQ and IRF4 and the other more ‘monocyte‐like’ showing CD14, CD32, CD36, CD163 and proportionately higher MAFB expression.^8^, ^9^ CD14^+^ CD1c^+^ cells have previously been detected and characterized as CD1c^+^ monocytes^139^ but by gene expression, most cluster with cDC2.^8^, ^9^ In tissues, especially during inflammation, and in humans affected by cancer, it is relatively easy to detect dual positive CD1c^+^ CD14^+^ cells^12^, ^13^, ^140^ but the origin of these may be difficult to ascertain precisely because as mo‐DC, they converge towards the monocyte‐like cDC2 phenotype.^30^, ^141^ Antibodies to CD2, CD5, Fc*ε*R1, CLEC4A (DCIR/CD367) and CLEC10A (CD301) may be useful but are still inducible and labile in inflammation.

### Development

Myeloid cDC2 are dependent on GATA2, PU.1, GFI1, ID2, ZEB2, RELB, IRF4, NOTCH2 and KLF4, but unlike pDC and cDC1, no single transcription factor has exclusive control over their development.^53^, ^60^, ^66^, ^142^ In mice, ZEB2 has recently been identified as a factor influencing the fate of pre‐DC towards the cDC2 lineage^66^ and IRF4 is considered to be a lineage‐defining factor.^2^ Depending upon the tissue site, subsets of murine IRF4^+^ cDC2 show variable dependence upon RELB, NOTCH2/RBPJ and lymphotoxin‐β or a requirement for KLF4.^60^ In humans, much less is known about the differential regulation of cDC2 production. Heterozygous GATA2 deficiency leads to eventual loss of all cDC2.^53^ Bi‐allelic IRF8 deficiency also abrogates cDC2 development because the entire monocyte‐DC tract of lympho‐myeloid development is lost.^61^, ^62^ In contrast, cDC2 are IRF8‐independent in mice,^100^ although great care is required to avoid inadvertently counting expanded primitive myeloid cells as cDC2 when both alleles of IRF8 are deleted. This may at least partly explain the phenotype of humans with heterozygous IRF8 T80A mutation, in which an abnormal population of CD11c^+^ CD1c^–^ cells appears in place of CD1c^+^ cDC2.^61^ Transcription factor zbtb46 is not required for development of cDC2 but is up‐regulated during differentiation and also appears on mo‐DC.^143^, ^144^

The heterogeneity of transcription factor dependence observed in mice is consistent with multiple subsets of cDC2, as recently described in humans.^8^, ^9^ IRF4, KLF4, LTBR and MAFB are slightly differentially expressed by cDC2 subsets but RELB and NOTCH2 are uniform across cDC2. There is no obvious correlation with pre‐DC or monocyte origin; pre‐cDC have high expression of both IRF4 and KLF4 whereas monocytes express comparable levels with DC of KLF4, MAFB, RELB and NOTCH2 (data from ref. 9).

### Functions and role in immunity

Myeloid cDC2 are equipped with a wide range of lectins, TLRs, NOD‐like receptors and RIG‐I‐like receptors similar in range to that expressed by monocytes. Human blood cDC2 respond well to lipopolysaccharide, flagellin, poly IC and R848.^145^ The potential of CD1c and CD1a to present the glycolipid antigens of mycobacteria and other pathogens is often overlooked.^146^ Among the lectins, CLEC4A (DCIR/CD367), CLEC10A (CD301) CLEC12A (CD371) and the asialoglycoprotein receptor are highly expressed. In common with monocytes, TLRs 2, 4, 5, 6 and 8 are present with notable expression of NOD2, NLRP1, NLRP3 and NAIP (data from ref. 9). Dectin‐1 (CLEC7A) and Dectin‐2 (CLEC6A) are highly expressed in tissue cDC2, suggesting a role in fungal recognition.^147^, ^148^ DEC205 (CD205; CLEC13B) and macrophage mannose receptor (CD206; CLEC13D) are variable.^95^

In contrast with mice, human cDC2 can be stimulated to become high producers of IL‐12 and excellent cross‐presenting cells.^20^, ^21^, ^107^, ^108^ Their ability to synthesize IL‐12 is greater than that of cDC1 in most conditions analysed. They secrete IL‐23, IL‐1, tumour necrosis factor‐*α* (TNF‐*α*), IL‐8 and IL‐10 but are consistently low in the secretion of type III interferon.^109^, ^149^ *In vitro*, human cDC2 are potent in the activation of Th1, Th2, Th17 and CD8^+^ T cells,^109^, ^149^, ^150^ suggesting the capacity to promote a wide range of immune responses *in vivo*. Subsets of cDC2 defined by CD5 and other markers differ in their production of TNF‐*α*, IL‐6, IL‐10 and IL‐23 in response to TLR ligation.^8^, ^140^ CD5‐high ‘DC‐like’ cDC2 are more active in CCR7‐dependent migration, stimulate high naive T‐cell proliferation and preferential priming of Th2, Th17, Th22 and regulatory T cells. ‘Monocyte‐like’ cDC2 with lower CD5 are less active in proliferation assays and produce mainly Th1 cells.^8^ Subsets of cDC2 specializing in Th2 or Th17 responses are dependent upon IRF4 or KLF4, respectively,^58^, ^60^ but this has not been corroborated in humans.

## Langerhans cells

### Phenotype and distribution

Langerhans cells are specialized DC that inhabit the basal epidermis and other stratified squamous epithelia. They express the C‐type lectin langerin and the invariant MHC class I molecule CD1a. The close integration of LC within the epithelial layer is mediated by E‐Cadherin, EpCAM (TROP1) TROP2, AXL and tight junction proteins claudin, occludin and ZO‐1.^151^, ^152^ In common with myeloid cDC2 they express high levels of Fc*ε*R1 and CD39 (ATPase). Myeloid cDC2 spontaneously express a low level of langerin in many sites, but the high expression of langerin, CD1a and EpCAM, together with lower expression of CD11c, CD11b and CD13 by LC, is usually sufficient to separate them clearly.^22^, ^131^ Care must be taken not to include cDC1 and LC together as both show lower CD11c expression than cDC2. This has led to controversy about the relationship of LC with the cDC1 lineage.^111^, ^153^ Although LC are distinct from cDC1 ontogenetically,^111^ it is clear that they do have functional cross‐presentation capacity^154^, ^155^ and high MHC class I‐related gene expression in humans.^111^ LC migrate to skin‐draining lymph nodes, where they appear in the T‐cell areas expressing langerin, CD1a and CD1c. EpCAM and other epithelial markers are down‐regulated, making it more difficult to distinguish LC from cDC2 by microscopy, although langerin is still expressed. Differences between inflamed and non‐inflamed skin‐draining nodes^156^ and between skin‐draining nodes and tonsil^22^ have been used to highlight the contribution of migratory LC to nodal DC populations.

### Development

Langerhans cells are phylogenetically ancient and share a primitive origin with tissue macrophages and microglia of the brain.^157^ This has led to their classification as ‘macrophages’,^1^ a controversial position given that they epitomize the function of myeloid DC that capture antigen, mature and migrate to lymph nodes.^158^ Recent lineage tracing experiments in mice indicate that LC have an equal claim to DC and macrophage heritage by virtue of unique dual expression of ZBTB46 and MAFB.^159^ Their development is unique among DC in that once established by primitive and fetal liver haematopoiesis, LC are capable of local self‐renewal, independently of the bone marrow.^160^ Proliferating human LC were described many years ago^161^ and self‐renewal may be demonstrated in humans lacking DC‐poiesis due to mutations in GATA2 or IRF8^62^ ^,162^ or recipients of limb transplantation.^163^ Recent data indicate that mucosal LC are more dependent upon bone‐marrow‐derived precursors.^164^

In mice, the establishment of a self‐renewing network is dependent upon PU.1 RUNX3 and ID2 in combination with locally produced cytokines IL‐34, TFG‐*β* and bone morphogenetic protein 7 (reviewed in ref. 165). The sequential formation of human fetal LC from myeloid precursors has been observed in detailed microscopic studies and ascribed to non‐monocyte precursors.^166^ In the steady state, the influence of TGF‐*β*, E‐cadherin/*β*‐catenin and the binding of Axl to gas 6 maintains LC in a quiescent state.^151^, ^152^ ^,167,168^

In postnatal life, local self‐renewal restores LC numbers following chronic or low‐grade inflammatory insults.^169^ However, studies in mice show that severe inflammation recruits *de novo* bone‐marrow‐derived precursors in two waves; a transient population of classical monocytes followed by uncharacterized myeloid precursors that form a stable self‐renewing LC network as inflammation subsides.^170,171^ Bone marrow transplantation in humans also results in replacement by donor cells even after non‐myeloablative treatment and in the absence of overt graft‐versus‐host disease.^172,173^

*In vitro* models of LC development from CD34^+^ progenitors, cDC2 and monocytes offer potential routes to explain the repopulation of LC *in vivo*, following severe inflammation.^174^ Purified monocytes, contrary to early reports, do not make LC efficiently with GM‐CSF and TGF‐*β*,^175^ but require additional notch signals to down‐regulate a KLF4‐dependent pathway of differentiation.^176–178^ Myeloid cDC2, however, rapidly up‐regulate langerin to high levels and form Birbeck granules with GM‐CSF or TSLP and TGF‐*β* or BMP7.^133^, ^134^ ^,179^ *In vivo* evidence for these two pathways has recently been reported in the homeostasis of mucosal LC^164^ but direct evidence is lacking in humans.

### Functions and role in immunity

When the skin becomes inflamed, local production of TNF‐*α* and IL‐1*β* stimulate LC to lose their connections with the surrounding epithelium and migrate across the basement membrane into the afferent lymphatics. Although LC were the primary model of migratory myeloid DC, their non‐redundant function in immunity has been surprisingly difficult to pin down. In humans, they can be matured into potent cross‐presenting DC with high IL‐15 production and the ability to present mycobacterial glycolipid antigens and stimulate CD8 T cells.^154^, ^155^ Transgenic expression of human CD1a on murine LC licensed them for presentation of lipid antigens to Th17 and Th22 cells. resulting in skin inflammation.^180^ However, it has also been reported that LC lack critical TLRs^145^ and can induce regulatory T cells and IL‐22 production through CD1a‐restricted antigens to autologous T cells.^181^ Overall, the role of LC has been summarized as maintaining epidermal health and tolerance to commensals, while retaining the ability to respond to selected intracellular pathogens and viruses under inflammatory conditions.^182^

## Monocyte‐derived inflammatory DC

### Phenotype and distribution

The term ‘inflammatory dendritic cell’ has come to be defined as monocyte‐derived DC (mo‐DC) that appear in inflammation. Ontogeny‐based nomenclature would require that the term monocyte‐derived is retained, since DC lineage cells may also be recruited in inflammation.^183^ In mice, inflammatory mo‐DC were originally demonstrated in leishmaniasis but have now been recognized in other infections and inflammatory settings (reviewed in refs. 184,185). In humans, inflammatory myeloid cells have been reported in several settings including eczema,^186^ psoriasis,^187,188^ skin sensitization,^189^ allergic rhinitis,^190^ coeliac disease,^191^ inflammatory bowel disease,^192,193^ synovitis and peritonitis.^24^ ^,194^ Kinetics, surface markers, gene expression and even direct labelling suggest that monocyte‐derived cells dominate these populations. Considerable heterogeneity is observed with no clear consensus on the use of the terms monocyte, macrophage or DC to describe inflammatory monocyte‐derived cells (Table 2). Inflammatory monocytes retain expression of CD13, CD33, CD11b, CD11c and CD172a and may show recent evidence of recruitment from monocytes by their expression of S100A8/9 and CCR2.^190,194^ In humans, monocytes express CD11c and MHC class II so these markers are not helpful in separating monocytes from DC. Evidence of DC differentiation is supported by the expression of CD1c, CD1a, Fc*ε*R1, IRF4 and ZBTB46. Inflammatory mo‐DC have also been described to express CD206 and CD209, but retained expression of CD14 and co‐expression of CD16, CD163 and FXIIIA, together with lack of CD1c, CD1a and FceR1, are more consistent with a phenotype usually described as macrophage‐like. In two studies, where it was possible to characterize dual populations of DC‐like and macrophage‐like cells, the key properties linked to DC phenotype were CD11c, CD1c, Fc*ε*R1, CD206, IRF4 and allo‐stimulation. CD14 was variable but CD16 and CD163 were negative.^24^ ^,194^ Although gene expression showed subset‐restricted patterns, there were many shared transcripts between populations designated as mo‐DC and those described as monocyte‐derived macrophages.

**Table 2:**
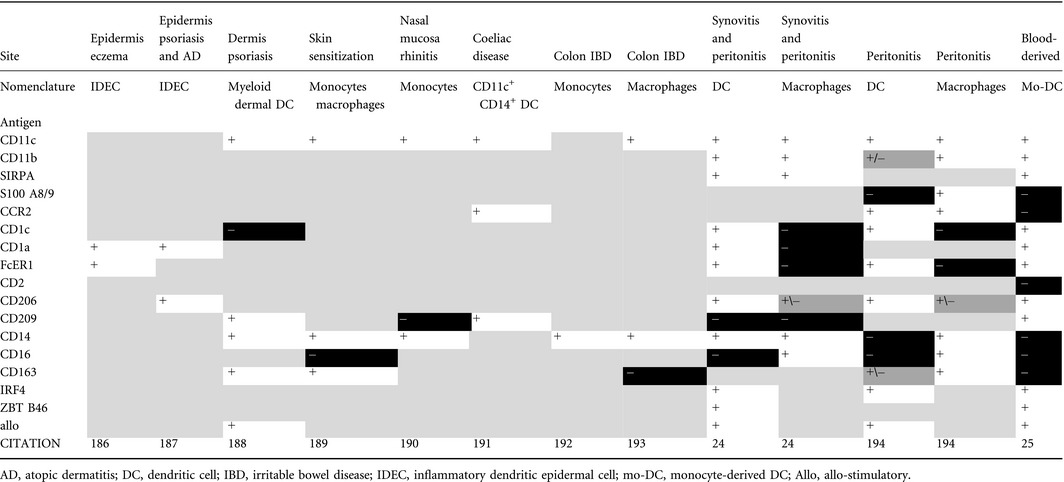
Features of monocyte‐derived dendritic cells

### Development and functions in immunity

Populations of monocyte‐derived cells exist in human steady‐state tissues, including the skin,^13^ lung^14^, ^15^, ^16^ and intestine.^17^ It is not known if this is a distinct mechanism of recruitment or one that is accelerated and activated in inflammation. During inflammation, monocyte‐derived cells expand resident populations many‐fold.^189–194^ Prevailing models suggest that monocyte‐derived cells function mainly at a site of inflammation rather than migrating to lymph nodes.^195^ However, mo‐DC studied *ex vivo* secrete IL‐1, TNF‐*α*, IL‐12 and IL‐23, stimulate CD4 and CD8 T cells and express CCR7.^24^ ^,188,194^ Although it is possible to derive potent mo‐DC *in vitro* with GM‐CSF and IL‐4 followed by activating stimuli such as lipopolysaccharide or prostaglandin E_2_, the use of such preparations in immunotherapy is declining in favour of ‘naturally‐occurring’ DC such as pDC or cDC2 (reviewed in ref. 196). The disappointing performance of mo‐DC in cancer immunotherapy is being interpreted as due to an intrinsic lack of biological potency of monocyte‐derived cells.^141^ However, the *in vivo* conditions under which mo‐DC form are still poorly replicated by *in vitro* experiments.

## CD16^+^ non‐classical monocytes, SLAN^+^ DC and DC4

The CD16^+^ non‐classical monocyte is still considered as a DC by some authors. In particular, those that express a carbohydrate modification of the P‐selectin glycoprotein ligand 1, SLAN (recognized by the antibody M‐DC8) have been identified as ‘SLAN DC’.^197^ The nomenclature is confusing however, as others have since used SLAN to identify a ‘true’ non‐classical monocyte population (distinct from intermediate CD14^+^ CD16^+^ cells).^198^ The simple facts are that CD16^+^ monocytes are heterogeneous and that SLAN expression identifies a subpopulation with lower CD11b, CD14 and CD36 but higher expression of CD16. Concerning the question of whether SLAN^+^ cells are monocytes or DC, their gene expression is clearly monocytic.^13^, ^198^, ^199^, ^200^ Furthermore, recent human *in vivo* and xenograft experiments support the hypothesis that non‐classical monocytes differentiate from classical monocytes by down‐regulating inflammatory pathways and adopting a CX3CR^+^ CCR2^–^ CD11c^hi^ CD11b^lo^ phenotype.^201^ However, it should be noted that gene expression alone does not absolutely exclude an independent origin of SLAN^+^ cells from the remainder of CD16^+^ monocytes and that SLAN itself was not measured on the output of cells derived from human classical monocytes in the adoptive transfer experiments.^201^

Most recently, single cell RNAseq studies found CD16^+^ cells among HLA‐DR^+^ lineage‐negative (CD3, CD19, CD56) CD14‐negative cells and classified them as ‘DC4’.^9^ DC4 was described as transcriptomically distinct from non‐classical monocytes, yet it was obtained from the same CD16^+^ population that contains non‐classical monocytes, by a slightly different gating route. One of the drawbacks of transcriptomic clustering is that it is difficult to relate population frequency back to input cells. The most likely explanation for DC4 is that is signifies a subset of CD16^+^ monocytes. Although it is possible to ‘index’ single cells back to their flow parameter space, the position of DC4 within the non‐classical monocyte gate was not shown. It is apparent that DC4 has a transcriptional profile reminiscent of SLAN^+^ cells with lower CD11b, CD14 and CD36 but higher expression of CD16. The expression of SLAN itself, a post‐translational modification, was naturally invisible to transcriptomic analysis. Recent experiments conclude that DC4 and SLAN^+^ cells are indeed identical^202^ but further studies are required to discover their non‐redundant roles in immunity.

## Conclusion

In summary, human DC arise by a dedicated pathway of lympho‐myeloid haematopoiesis and differentiate into specialized subsets under the influence of lineage‐specific transcription factors, notably IRF8 and IRF4. Plasmacytoid DC and cDC1 are the most specialized with relatively restricted roles in sensing nucleic acid and responding by interferon production. Cross‐presentation to CD8 cells is not restricted to cDC1 but can be performed by cDC2 and mo‐DC with appropriate activation. The wide range of CD4^+^ T‐cell priming capacity by cDC2 is likely to reflect heterogeneity within this population. Single‐cell experiments have clarified that pDC and pre‐cDC have often been previously analysed as a mixed population. DC subset specialization increases the range and flexibility of immune responses through mechanisms that are relatively conserved across mammalian species. This knowledge has become essential in translating murine immunology to human pathology and continues to expand the therapeutic horizon of DC in medicine.

## Disclosures

The authors have no competing interests to declare.
